# Multifaceted phytogenic silver nanoparticles by an insectivorous plant *Drosera spatulata* Labill var. bakoensis and its potential therapeutic applications

**DOI:** 10.1038/s41598-021-01281-8

**Published:** 2021-11-09

**Authors:** Susmila Aparna Gaddam, Venkata Subbaiah Kotakadi, Gunasekhar Kalavakunta Subramanyam, Josthna Penchalaneni, Varadarajulu Naidu Challagundla, Sai Gopal DVR, Visweswara Rao Pasupuleti

**Affiliations:** 1 Department of Virology, Sri Venkateswara University, Tirupati, Andhra Pradesh India; 2 DST-PURSE Centre, Sri Venkateswara University, Tirupati, Andhra Pradesh India; 3 Department of Biotechnology, Dravidian University, Kuppam, Andhra Pradesh India; 4 Department of Biotechnology, Sri Padmavathi Mahila Visvavidyalayam (Women’s University), Tirupati, Andhra Pradesh India; 5 Department of Biomedical Sciences and Therapeutics, Universiti Malaysia Sabah, Kota Kinabalu, Sabah Malaysia; 6 Department of Biochemistry, Faculty of Medicine and Health Sciences, Abdurrab University, Jl Riau Ujung No. 73, Pekanbaru, 28292 Riau Indonesia; 7 Centre for Excellence in Biomaterials Engineering (CoEBE), AIMST University, 08100 Bedong, Kedah Malaysia

**Keywords:** Biochemistry, Nanoscience and technology

## Abstract

The current investigation highlights the green synthesis of silver nanoparticles (AgNPs) by the insectivorous plant *Drosera spatulata *Labill var. bakoensis, which is the first of its kind. The biosynthesized nanoparticles revealed a UV visible surface plasmon resonance (SPR) band at 427 nm. The natural phytoconstituents which reduce the monovalent silver were identified by FTIR. The particle size of the Ds-AgNPs was detected by the Nanoparticle size analyzer confirms that the average size of nanoparticles was around 23 ± 2 nm. Ds-AgNPs exhibit high stability because of its high negative zeta potential (− 34.1 mV). AFM studies also revealed that the Ds-AgNPs were spherical in shape and average size ranges from 10 to 20 ± 5 nm. TEM analysis also revealed that the average size of Ds-AgNPs was also around 21 ± 4 nm and the shape is roughly spherical and well dispersed. The crystal nature of Ds-AgNPs was detected as a face-centered cube by the XRD analysis. Furthermore, studies on antibacterial and antifungal activities manifested outstanding antimicrobial activities of Ds-AgNPs compared with standard antibiotic Amoxyclav. In addition, demonstration of superior free radical scavenging efficacy coupled with potential in vitro cytotoxic significance on Human colon cancer cell lines (HT-29) suggests that the Ds-AgNPs attain excellent multifunctional therapeutic applications.

## Introduction

Presently, many researchers in the life science and biomedical field are working with scientists in material sciences to discover the various opportunities to detect the use of nanomaterials as innovative tools for cancer therapy, antimicrobials, wound healing and other industrial and pharmaceutical applications. Green synthesized silver nanoparticles (AgNPs) have great prospects in cancer theranostics, imaging, drug delivery, cell toxicity and DNA and protein binding and X ray biomedical applications and etc.^1–5^. It’s well known that metal nanoparticles having size 1 nm to 100 nm are of biomedical importance. Several reports on green synthesis yield metal nanoparticles with size less than 50 nm which are highly therapeutic in nature. So, green synthesis of AgNPs is presently a very much attentive research field due to its easy, fast, and eco-friendly method without involving the utilization of organic solvents and perilous chemicals and outlay to be efficient when compared with chemical and physical methods^6,7^. In addition, the plant based green synthesis is the finest candidate for wide-ranging fabrication of nanoparticles with controlled shape and size, however green synthesized nanoparticles comprise and detected to be irregular in size and shape in majority of the cases^8,9^. The bioactive phyto-constituents like polyphenols and other bio-constituents like flavinoids, tannins, alkaloids, saponins and glycosides, found in various parts of plants leaves, fruits, seeds, stem, bark and roots, are extensively used for biosynthesis of metal nanoparticles, which actively participate in reduction and stability of nanoparticles. The earlier reports from our studies also revealed that synthesized silver nanoparticles at room temperature are spherical and polydispered in nature, but anomalous in size and shape ranging between 20 and 80 nm, all these metal nanoparticles exhibit excellent properties like antioxidant, antibacterial, antifungal, anti-diabetic, anticancer, catalytic and other important biomedical applications^10–14^. There are several studies that have reported that plant bioactive compounds exert potential biomedical applications. These plant bioactive components used for the biosynthesis of green metal nanoparticles can show the new ways for the progress of novel strategies for the treatment of various cancers due to their synergistic effect^15–17^.

*Drosera* sp., commonly known as Sundews, was found to be an important medicinal herb since twelfth century. It has been widely used and recommended by herbalists to cure various ailments like asthma, bronchitis, bronchial cramps, dry coughs whooping cough^18^ and also exhibits anti-tussive properties^19^. It is also used as a natural aphrodisiac and to maintain the heart strength^20^. In addition, it is used in treating lung infections and stomach ulcer. *Drosera* sp. is carnivorous in nature and the beauty of the plants is due to glossy traps, as a result they have turn out to be much loved ornamental plants^21^. Most popular *Drosera* species consist of *D. aliciae*, *D. capensis* and *D. spatulata*^22^ next to Venus fly traps.

In recent times scientists are very much interested in checking the elastic properties of the mucilage produced by *Drosera* species like *D.* *binata*, *D. capensis*, and *D.* *spatulata,* which is an exceptionally smart idea for biomaterial research on synthesis of nanofibers and nanoparticles^23^. They have observed various sizes nanofibers and nanoparticles networks within the mucilage of these plants, which were confirmed by different spectroscopic techniques like Transmission electron microscopy (TEM) and Atomic force microscopy (AFM). The Energy-dispersive X-ray spectroscopy (EDAX) analysis revealed key biological salts like calcium, magnesium and chlorine^23^. The scientists detected a wide range of applications from Drosera *mucin or mucilage*, which includes wound healing properties, tissue engineering and regenerative medicine. The mucilage of *Drosera* species is readily available source of biomaterial due its stretching property which can stretch million times then its original size^23^. Sundews also contains several potential biologically active chemical compounds like flavoniods They also contain additional important phyto-constituents like carotenoids and various plant acids, resins, tannins and very important vitamin like ascorbic acid (vitamin C)^24–26^.

The present work is primarily focused on the green synthesis of AgNPs using insectivorous plant extract of *Drosera spatulata *Labill var. bakoensis, which acts both as a reducing and stabilizing agent. The synthesized green Ds-AgNPs were used for subsequent spectral characterization using UV–vis, FTIR, XRD, TEM, EDAX and AFM. They were further used to explore the antioxidant, antimicrobial studies, and anticancer activities against human colon cancer cells to detect their potential therapeutic applications in different areas of biomedical sciences.

## Materials and methods

### Collection of plant material and preparation of the extract

**Figure 1:**
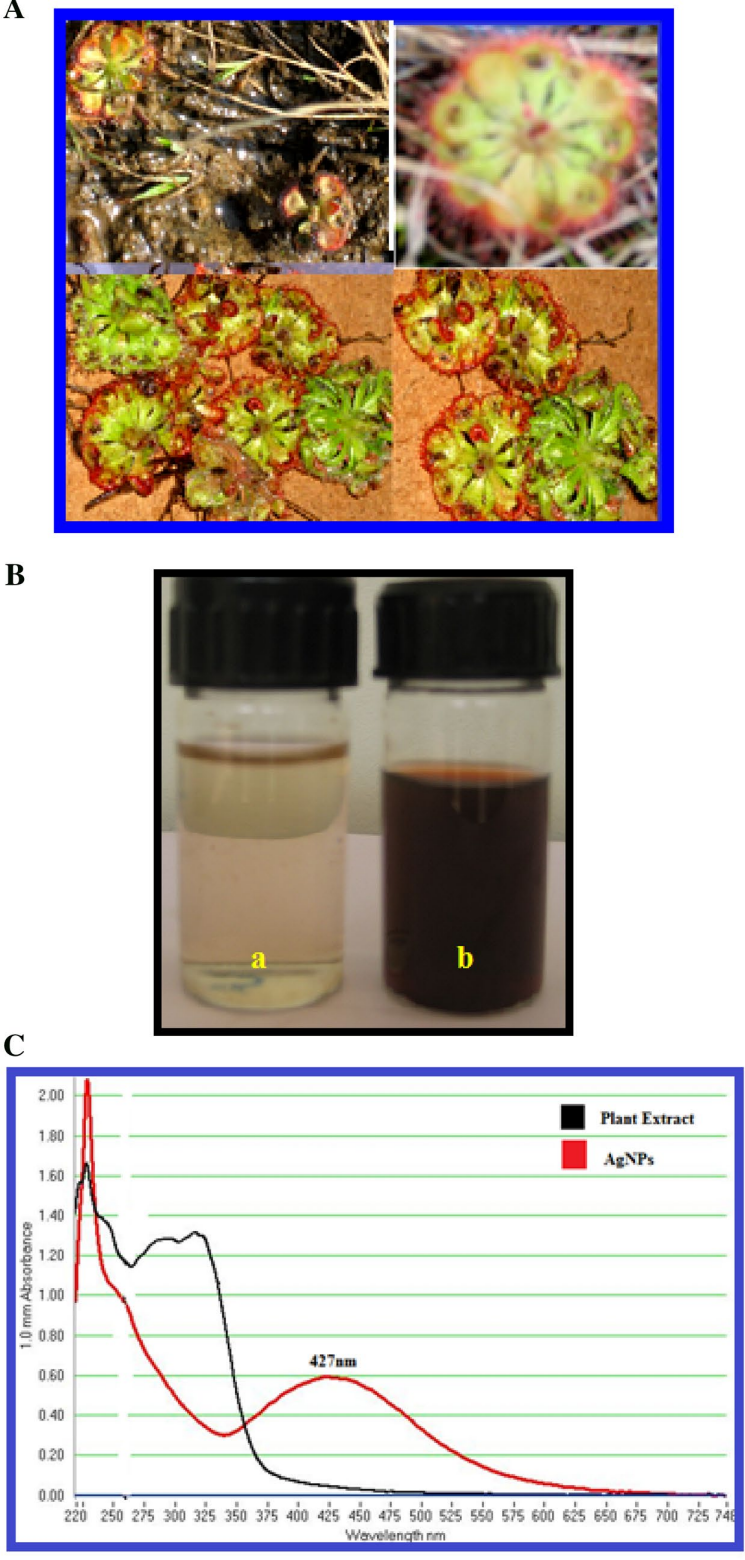
(**A**) *Drosera spatulata* plant. (**B**): (a) Plant extract of *Drosera spatulata*. (b). Biosynthesized Ds-AgNPs by Plant extract of *Drosera spatulata*. (**C**): UV–VIS absorption spectra of *Drosera spatulata* plant extract (black spectrum) and UV–VIS absorption spectra of Ds-AgNps synthesized from ***Drosera spatulata*** plant extract with 0.002 M silver nitrate (red spectrum).

*Drosera spatulata *Labill var. bakoensis A.Fleischm.&Chi C.Lee plants (Fig. 1A) were collected from the nitrogen deficient lands located at Thottambedu, Srikalahasti, Chittor district, Andhra Pradesh. (12°37′–14°8′ north latitudes and 78°3′–79°55′ east longitudes). The identified plant was confirmed and authenticated [Herbarium No.NN2120/PURSESVU/2021] by Dr. N.Nagaraju, Associate Professor and Head Retd, Department of Botany, Sri Venkateswara Arts and Science College, Tiruapti, Andhra Pradesh and a voucher specimen has been deposited in the department. The plant height was 2 cm, the root depth was 0.50 cm and it was highly associated with soil particles while the plant flowered part diameter measured was 1.8 cm. Wild *D. spatulata *var. bakoensis plant materials were collected from the place which was the open space and belong to the private land, which the main author has obtained oral permission from them. All the methods used in plant collection and authentication was appropriate with standard guidelines. There are no unethical practices involved in any of the methods in plant collection and preparations. The plant materials were thoroughly washed with distilled water until the roots were completely dissociated from the soil particles. Then these plants were dried in the shade at room temperature for 6 to 9 days and the dried plant material is kept in the hot air oven at 40 °C temperature for overnight to remove any moisture left in the sample. The dried plant samples were ground into fine powder which was brownish in color and was used to carry out further studies. The collection of plant material was made with relevant institutional, national, and international guidelines and legislation. The Fig. 1A photographs were taken by the author and are not copied from any other sources.

### Green synthesis of silver nanoparticles

The *D. spatulata* var. bakoensis plants extract was prepared by taking 5 g of fine plant powder with 250 mL of Milli Q water in a sterile 500 mL conical flask and steadily mixed the mixture solution and kept in water bath at 60 °C for 30 min, soon after than 30 min the prepared extract was filtered through sterile muslin cloth followed by Whatman no.1 filter paper. The filtrate solution was used to carry out the green synthesis of silver nanoparticles. 5 mL of plant extract was diluted to 20 mL with sterile Milli Q water and then added 50 mL of 0.002 M AgNO_3_ solution_._ The reaction mixture was left at room temperature and observe for color change of the solution, after few minutes the color changed to pale brown and subsequently to dark brown color within 10 min (Fig. 1Ba,b). The color change of the reaction solution is due to the reduction of AgNO_3_ to silver ions. In the current study, the AgNPs have been biosynthesized by using whole plant extract of *D. spatulata *var. bakoensis without any toxic chemicals. Hence this method is an environmentally safe "Green method”; hence the biosynthesized nanoparticles can be referred as Ds-AgNPs.

### Purification of biosynthesized silver nanoparticles or green *Ds-*AgNPs

The purification Ds-AgNPs was carried out with modified procedure of Sucharitha et al.^40^. The biosynthesized Ds-AgNPs solution containing silver nanoparticles were centrifuged at 15,000 rpm for 30 min to obtain the pure Ds*-*AgNPs pellet. The Ds*-*AgNPs pellets were re-dispersed in 15 mL of sterile Milli Q water to get rid of unbound plant extract residues and biological molecules. The process of centrifugation and re-dispersion of Ds-AgNPs in sterile Milli Q water was repeated thrice to obtain pure green Ds*-*AgNPs. The purified green Ds*-*AgNPs pellets were used for consecutive studies like FTIR, EDX, XRD and other advanced spectroscopic methods like TEM and AFM.

### Spectral characterization of Ds-AgNPs

The biosynthesized Ds-AgNPs by the total plant extract of *D. spatulata *var. bakoensis was analyzed using Nanodrop 8000 (UV–visible spectrophotometer, Thermo Scientific). The optical absorbance of the Ds-AgNPs was recorded at 220–750 nm wavelength range from time-to-time sampling 1 µl of sample and the reading were carried out at room temperature with 1 nm resolution^54^. The Fourier transformed infrared (FTIR) spectrum analysis was carried out for biosynthesized Ds-AgNPs using Alpha T model, FTIR spectrophotometer, Bruker Company, to reveal the potential bioactive components of plant extract of *D. spatulata *var. bakoensis responsible for the bio-reduction and stabilization of Ds-AgNPs. The particle size and zeta potential of the biosynthesized nanoparticles were determined by using Dynamic light scattering technique on Nanopartica analyzer SZ-100, Horiba scientific, Japan. The size, exterior surface morphology and topology of the green synthesized Ds-AgNPs were analyzed using Atomic Force Microscope (AFM-Solver Next, NT-MDT, Russia), by coating a thin film of Ds-AgNPs on sterile glass piece and air-dried completely prior to analysis. Transmission electron microscopy (TEM) analysis was carried out to identify the exact size and morphology of the synthesized Ds-AgNPs. The sample for TEM analysis was prepared by placing a drop of the purified Ds-AgNPs solution on a carbon-coated copper grids and allowed to dry completely and TEM analysis was carried out using FEI Tecnai F12 (Philips Optics Ltd, Holland) operated at 100 kV^38^ TEM was also used to study the selected area electron diffraction (SAED) pattern of the Ds-AgNPs and to get best SAED pattern, the image mode was maintained by Z control (sample height) while the objective lens and standard current were kept. The diffraction spots and the size of the Ds-AgNPs were determined using SIS imaging software (Munster, Germany). X-Ray Diffractometry (XRD) analysis was done to verify the crystalline nature of the Ds-AgNPs using Cu^2+^ Kα radiation source on an Ultima IV X-ray powder diffractometer (Rigaku Ltd, Tokyo, Japan). Energy dispersive X-ray (EDAX) was also performed by Oxford Inca Penta FeTX3 EDS instrument attached to Carl Zeiss EVO MA 15 Scanning Electron Microscope (200 kV) machine with a line resolution 2.32 (in Å). The analysis was done by coating a drop of purified Ds-AgNPs on an aluminum foil.

### In vitro antioxidant activity

#### Free radical scavenging activity of Ds-AgNPs by DPPH method

Free radical scavenging activity of the Ds-AgNPs was determined by using 2,2′-diphenyl-1-picrylhydrazyl (DPPH) radical scavenging assay according to the method described by Mittal et al.^27^. The DPPH stock solution was prepared by dissolving 4 mg of DPPH in100 mL of methanol and stored at 20 °C. DPPH solution (2 mL) was added to 1 mL of methanol solution containing test samples of *D. spatulata *var. bakoensis plant extract and biosynthesized Ds-AgNPs at different concentrations of 25 μg/mL, 50 μg/mL, 75 μg/mL and 100 μg/mL. The radical scavenging activity (RSA) was calculated by determining the absorbance at 517 nm, by using standard ascorbic acid, the antioxidant activity was expressed as IC_50_. The IC_50_ is the half maximum inhibitory concentration of any substance inhibiting a specific biological process or function.

$${\text{RSA }}\left( \% \right)\, = \,[({\text{control absorbance}} - {\text{sample absorbance}})/\left( {\text{control absorbance}} \right)] \times 100.$$

#### Hydrogen peroxide (H_2_O_2_) scavenging activity

H_2_O_2_ scavenging ability of the *D. spatulata *var. bakoensis plant extract and biosynthesized DsAgNPs at different concentrations of 25 μg/mL, 50 μg/mL, 75 μg/mL and 100 μg/mL, was examined according to the modified method of Pick and Mizel^28^. H_2_O_2_ (40 mM) solution was prepared in phosphate buffer (pH 7.4), Ds-AgNPs and plant extract at different concentrations of 25 μg/mL, 50 μg/mL, 75 μg/mL and 100 µg/mL in 3.4 mL phosphate buffer were added to H_2_O_2_ solution (0.6 mL, 40 mM). The absorbance of the reaction mixture was recorded at 230 nm and the percent of scavenging activity of H_2_O_2_ was determined using the equation as defined for DPPH scavenging activity.

#### Nitric oxide (NO) scavenging activity

The Nitric oxide scavenging activity was carried out by a tailored method of Ferreira et al.^27^. Nitric oxide radicals (NO) were generated from sodium nitroprusside. Sodium nitroprusside ( 1 mL of 10 mM) and 1.5 mL of phosphate buffer saline (0.2 M, pH 7.4) were added to different concentrations (25, 50, 75 and 100 µg/mL) of the Ds-AgNPs and plant extract of *D. spatulata *var. bakoensis and incubated for 150 min at 25 °C. The reaction mixture (1 mL) was treated with 1 mL of Griess reagent (1% sulfanilamide, 2% H_3_PO_4_ and 0.1% naphthylethylenediamine dihydrochloride). The absorbance of the reaction mixture was measured at 546 nm and nitric oxide scavenging activity was calculated using the equation as defined for DPPH scavenging activity.

### Antimicrobial activity

#### Antibacterial activity

The antibacterial activity of Ds-AgNPs was evaluated against three different strains of Gram negative *Escherichia coli* as follows: *E. coli* Strain-I (Donor) rifampin resistant, *E. coli* AB1157 (Mutant) streptomycin resistant and *E. coli* (Recipient strain) streptomycin resistant by the disc diffusion method according to modified protocol Kotakadi et al.^11^. Fresh bacterial cultures were prepared by transferring single colony of respective bacterial culture into a tubes containing 20 mL nutrient broth separately (Himedia, gm/L) and cultured overnight at 37 °C in an incubator come shaker. Three replicates of individual microorganisms were prepared by spreading 200 μl of culture on the nutrient agar plate with the help of sterile glass spreader. Discs were prepared by using Whatman No.1 filter paper. The discs were placed on agar plates and sample plant extract of *D. spatulata *var. bakoensis 30 μl and biosynthesized Ds-AgNPs 15 μl and 30 μl (μl = mcg) were added on the disc with the help of micropipette. Amoxyclav (Himedia SD063, 30 mcg) disc was used as reference drug. The plates were incubated at 37 °C overnight in an bacteriological incubator. The zones of inhibition (ZOI) of Ds-AgNPs along with standard drug were measured and tabulated.

#### Antifungal activity

The antifungal activity of Ds-AgNPs was evaluated against two fungal species *Aspergillus niger* and *Penicillium* sp. at concentration of 25 mcg by disc diffusion method along with the reference drug Nystatin, (SD025, HiMedia). The ZOI of Ds-AgNPs, ZOI of standard drug Nystatin, (SD025, HiMedia) and the ZOI of plant extract of *D. spatulata *var. bakoensis and 0.002 M silver nitrate solution were analyzed, and the results were tabulated.

### Cell culture

#### MTT in vitro assay for cytotoxicity of Ds-AgNPs

The cytotoxic activity of biosynthesized Ds-AgNPs was measured by using MTT [3-(4,5-dimethylthiazol-2-yl)-2,5-diphenyl tetrazolium bromide assay^29^. Human colon cancer cell lines (HT-29) were obtained from the National Centre for Cellular Sciences, Pune, India. Cells were cultured in Eagle's Minimum Essential Medium, supplemented with 10% fetal bovine serum, 2 mM glutamine, 1 mM NaHCO_3_, 100 μg/mL streptomycin and 100 units/mL penicillin. The cell lines were maintained at 37 °C in presence of 5% CO_2_ atmosphere in an incubator. The cytotoxic assay was carried out by our previous protocol explained in recent research articles^35,36,40^ with different concentration of Ds-AgNPs. Approximately 2 × 10^4^ HT-29 colon cancer cells were seeded in each well of 96 well culture plate containing 100 µL of medium. After overnight incubation, exactly 100 µL of Ds-AgNPs at different concentrations (0, 12.5, 25, 50, 100 and 200 µg/mL) were added to the cell suspension and incubated for 24 h. After 24 h of incubation, the viability of cells was assessed by adding 10μL of MTT (5 mg/mL) per well and incubated at 37 °C for additional 3 h. The 96 well plates were centrifuged at 1000*x*g for 10 min at room temperature. Formazan blue that formed in the cells was dissolved using 100 µL of DMSO. The intensity of color formation was measured at 570 nm wavelength. The percentage of inhibition of cell viability was determined and the IC_50_ concentrations were calculated.

## Results and discussion

### Spectral characterization of *Ds*-AgNPs

#### UV–visible spectral analysis

UV–Vis spectroscopy is the key method recommended to find out the possible characteristics of biofabricated silver nanoparticles (AgNPs). In the current investigation, color less plant extract of *D. spatulata *var. bakoensis color was changed to pale reddish brown and later to murky brown in color within couple of minutes after addition of 0.002 M silver nitrate solution shown in Fig. 1Ba,b. The above reaction clearly proves that the plant extract consists of several bioactive components which have a very good potential in the reduction of 0.002 M silver nitrate into silver nanoparticles i.e. Ds-AgNPs. The SPR spectrum of biosynthesized Ds-AgNPs was detected at 427 nm (Fig. 1C, Ds-Plant Extract, Ds-AgNPs). The metallic silver nanoparticles have SPR region in-between 390 and 470 nm. Earlier reports reveal that the AgNPs having the SPR spectrum in the region in-between 390–420 nm and 410–450 nm revealed to have small size nanoparticles around 25–50 nm and spherical in shape^30,31^. The plant bioactive compounds such as flavanoids, phenols, alkaloids and sugars, etc.are responsible for capping and stabilizing of metal nanoparticle by using biological extracts^32^.

#### FTIR analysis of biosynthesized Ds-AgNPs

**Figure 2:**
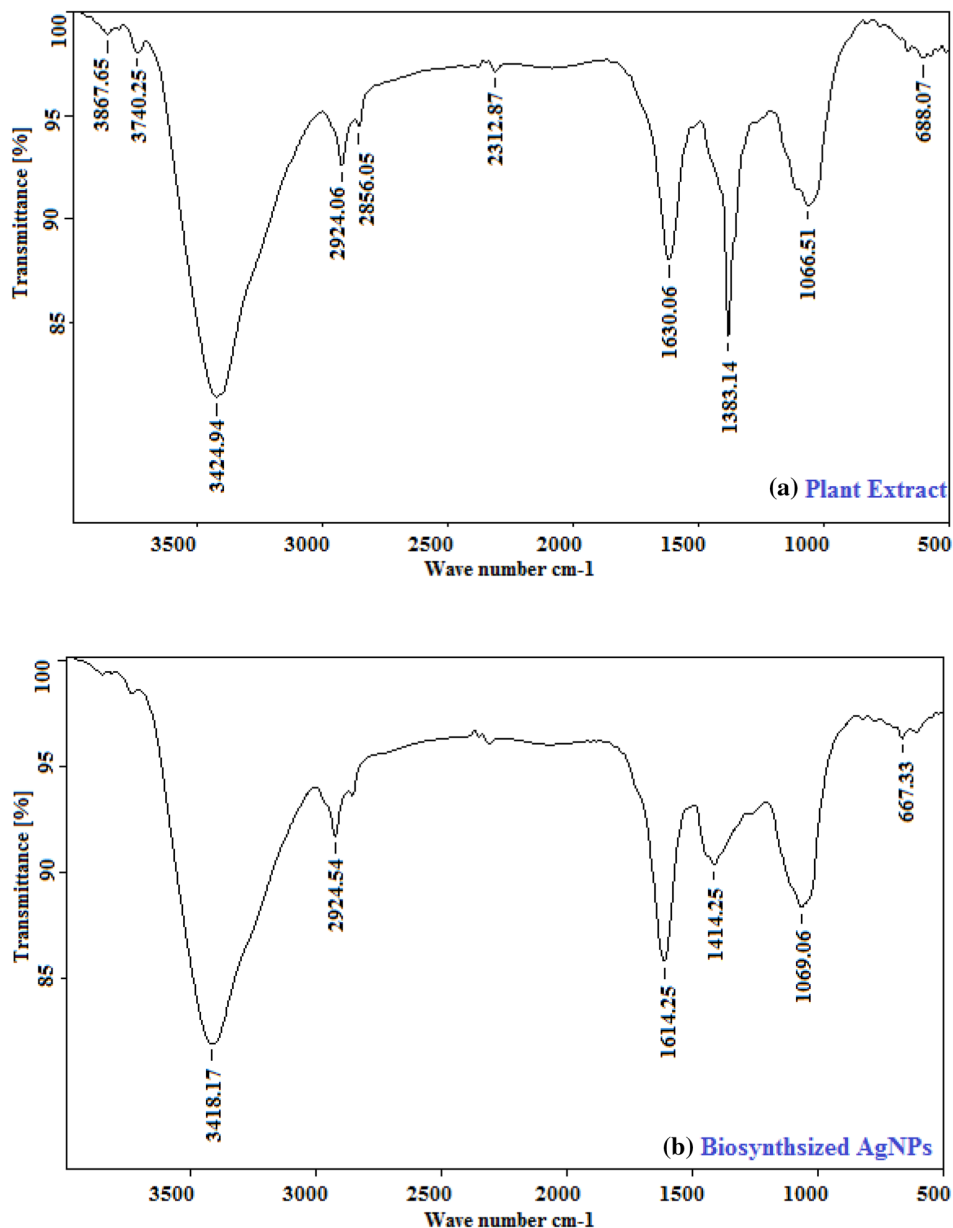
(**a**) FTIR spectrum of the *Drosera spatulata* plant extract. (**b**) FTIR spectrum of the biosynthesized Ds-AgNPs of *Drosera spatulata* plant extract.

FTIR analysis of *D. spatulata *var. bakoensis plant extract and Ds-AgNPs were analyzed separately to investigate the functional groups responsible for bio-reduction and stabilization of Ds-AgNPs and the results are shown in Fig. 2a,b. The FTIR spectra of plant extract showing the following functional peaks**;** 3867.65 cm^−1^, 3740.25 cm^−1^, 3424.94 cm^−1^, 2924.06 cm^−1^, 2856.05 cm^−1^, 2312.87 cm^−1^, 1630.06 cm^−1^, 1383.14 cm^−1^, 1066.51 cm^−1^ and 688.07 cm^−1^ were observed and FTIR spectra of biosynthesized Ds-AgNPs revealed the following functional groups**;** 3418.17 cm^−1^, 2924.54 cm^−1^, 1614.25 cm^−1^, 1414.25 cm^−1^, 1069.06 cm^−1^ and 667.33 cm^−1^. The functional groups of biomolecules were identified after bio-reduction of Ag^+^ to silver nanoparticles i.e. Ds-AgNPs as capping and stabilizing molecules. The functional groups in plant extract of *D. spatulata *var. bakoensis at 3424 cm^−1^ corresponds to O–H stretching of vibrations, indicating the existence of alcohols and phenols while peaks at 2924.06 cm^−1^ and 2856.05 cm^−1^ belong to C–H region arising from the stretching and bending of aromatic compounds. The peak at 2312.87 cm^−1^ corresponds to C–H stretching of methyl, methylene groups and methoxy groups, while 1630.06 cm^−1^ due to the stretching of C-N and C–C, indicating the presence of protein. Also, 1383.14 cm^−1^ belongs to typical stretching of N=O, which corresponds to nitro compounds, 1066.51 cm^−1^ corresponds to C–N vibration stretches of amines, which belongs to protein and 667.33 cm^−1^ belongs to C–H bending and C=C–H stretching vibrations of amides. Whereas the functional groups of Ds-AgNPs at 3418.17 cm^−1^ corresponds to O–H stretching indicating presence of alcohols and Phenols while peaks at 2924.54 cm^−1^, 1614.25 cm^−1^ due to the stretching of C–N and C–C indicating the presence of proteins. The peaks at 1414.25 cm^−1^ may be due to vibration stretches of =CH-H belongs alkanes and C–O/C–H bending of alkanes. Also 1069.06 cm^−1^ corresponds to C–N vibration stretches of amines which belong to proteins and 667.33 cm^−1^ belongs to C–H bending C≡C–H stretching vibrations of amides. So**,** from the above results it can be concluded that O–H stretching of alcohols and Phenols, C–N and C–C of the proteins, =CH–H, C–O/C–H bending of alkanes, N=O which corresponds to nitro compounds and C–H bending C≡C–H stretching vibrations of amides present in the plant extract might be responsible for the bio-reduction of Ag^+^ to AgNPs i.e. Ds-AgNPs. Similar results were reported on FTIR analysis by biosynthesized silver nanoparticles by other plant resources^33–36^.

#### Particle size and zeta potential of biosynthesized Ds-AgNPs

**Figure 3:**
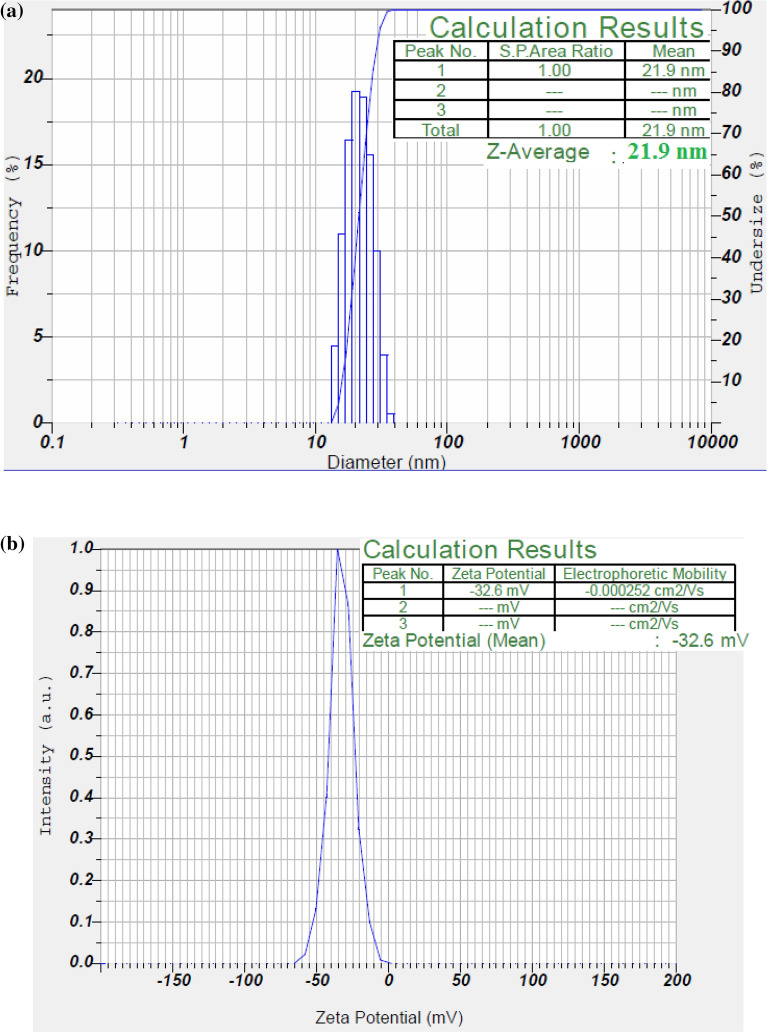
(a) Particle size distribution curve for Ds-AgNPs. (**b**) Zeta potential of synthesized Ds-AgNPs.

The particle **s**ize and zeta potential of the biosynthesized Ds-AgNPs was measured using dynamic light scattering (DLS) method to determine the size distribution and electrical charge present on the surface of Ds-AgNPs. Our results revealed that the size distribution of biosynthesized Ds-AgNPs was between 15 and 40 nm with an average size of 21.9 nm (Fig. 3a) indicating that biosynthesized Ds-AgNPs are poly dispersed in nature. The zeta potential of Ds-AgNPs was detected to be − 32.6 mV (Fig. 3b), due to its high negative potential the nanoparticles are well dispersed in biosynthesized colloidal solution. The stability of the Ds-AgNPs was due to their similar charges on the surface which will oppose the agglomeration. When the zeta potential is lower the attraction between nanoparticles exceeds repulsion and promotes aggregation of the nanoparticles. The zeta potential of silver nanoparticles using other plants also exhibit similar type of results^37,38^. Therefore, the Ds-AgNPs are highly stable and well dispersed in the biosynthesized colloidal solution.

#### Transmission electron microscopic analysis of biosynthesized Ds-AgNPs

**Figure 4:**
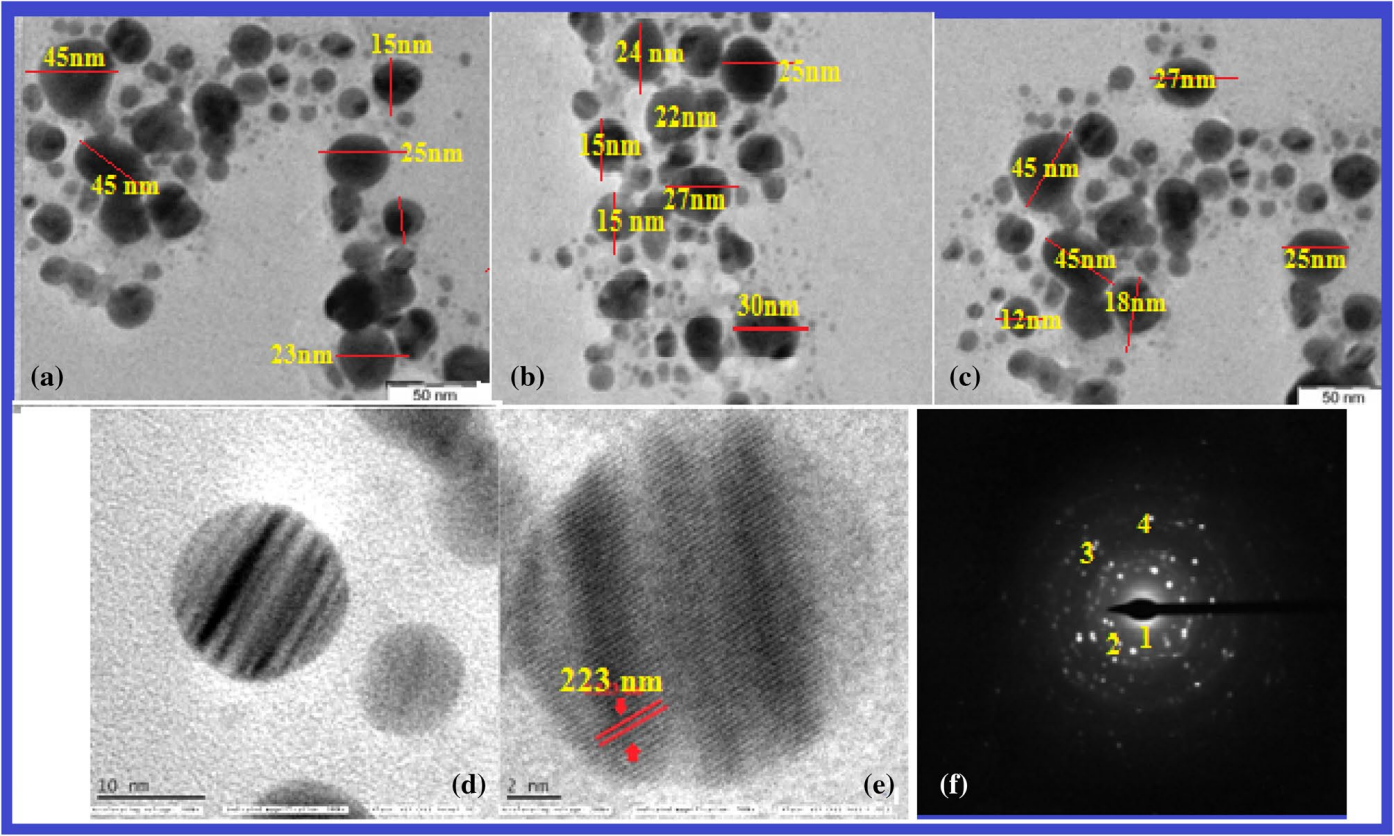
TEM images of Ds-AgNPs at magnification of (**a**) 50 Kx, (**b**)50 Kx, (**c**) 50Kx, (**d**) 280Kx, (**e**) 700Kx, (**f**) SAED pattern showed four diffraction rings.

TEM analysis provides the images of nanoparticles, and these images are helpful to find out the size, shape, surface morphology, texture, and distribution of nanoparticles. The results revealed that the Ds-AgNPs were roughly sphere-shaped with variations in size distribution, The bio-nanoparticles Ds-AgNPs are in the range from 5 to 40 nm at different magnifications (Fig. 4a–d). The Ds-AgNPs TEM images revealed that the nanoparticles are poly dispersed in nature without any agglomeration. The variation in size and shape of biosynthesized nanoparticles may be due to the presence of bioactive molecules of the plant extract present on the surface of the Ds-AgNPs. The Ds-AgNPs crystal analysis at 2 nm resolution revealed the crystal lattice fringes with the d spacing value of 0.223 nm (Fig. 4e). Later on, the crystal analysis done by SAED pattern (Fig. 4f) showed evidently Debye-Scherer rings of different planes of face centric cubic structures. With the above results it is concluded that the biosynthesized Ds-AgNPs are crystalline in nature, the results were similar to the earlier reports of biosynthesized AgNPs by leaf extracts of *Andrographis serpyllifolia* and also fruit extract of *Terminalia belerica*^39,40^.

#### XRD analysis of biosynthesized Ds-AgNPs

**Figure 5:**
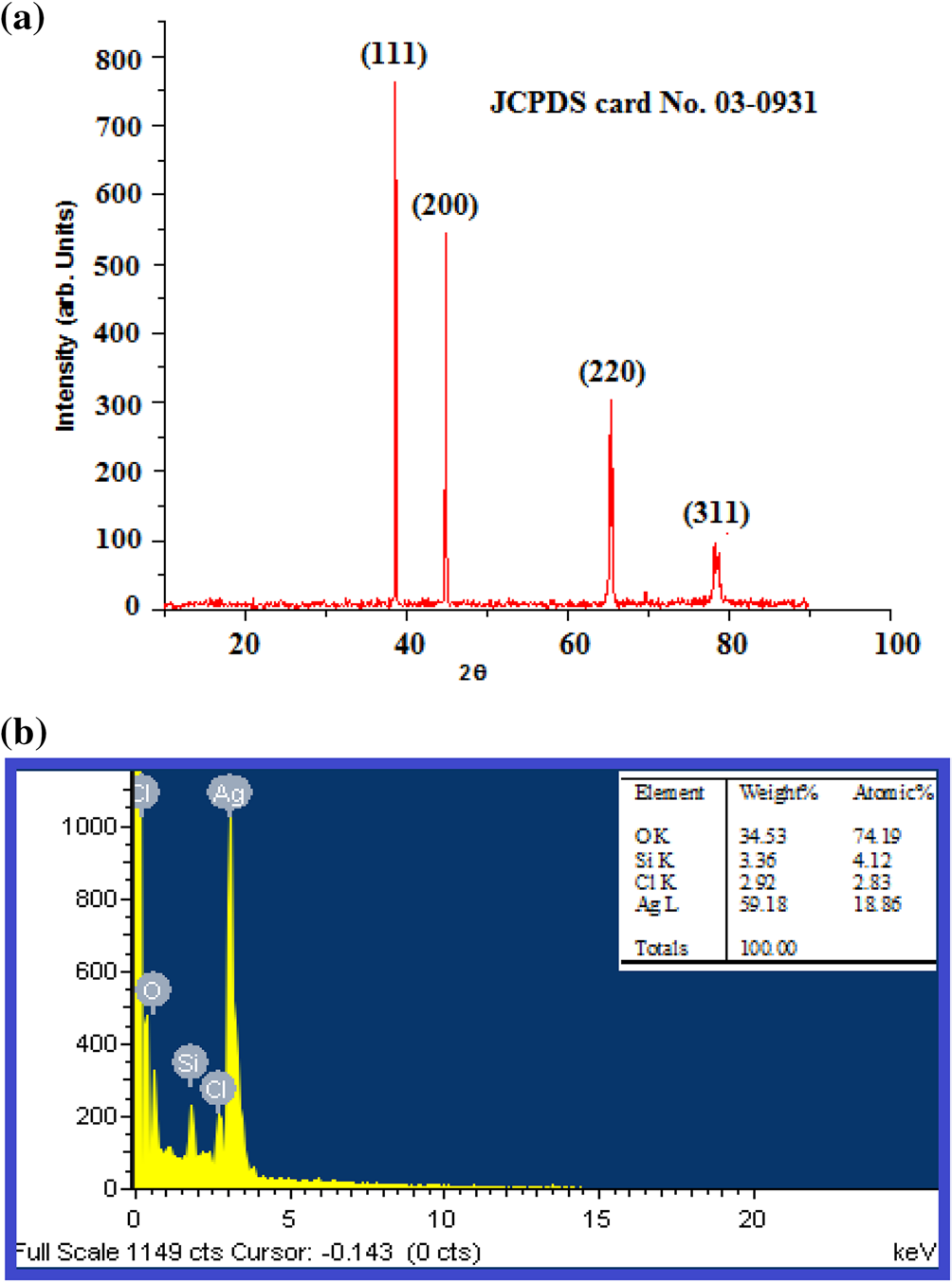
(**a**) XRD spectral data of biosynthesized Ds-AgNPs. (**b**) EDX analysis of fabricated Ds-AgNPs by plant extract of *Drosera spatulata.*

X-ray diffraction analysis of Ds-AgNPs was done to find out the crystalline characteristics of nanoparticles (Fig. 5a). The spectral analysis of the Ds-AgNPs revealed diffraction peaks at 38.7°, 44.9°, 65.3° and 78.4° and were respectively indexed to planes of face centered cubic (FCC) crystal lattice (111) (200) (220) and (311). The XRD outcome is consistent with standard JCPDS data (JCPDS No.03- 0931).

#### Energy dispersive X-ray spectroscopy (EDX) analysis of biosynthesized Ds-AgNPs

The EDX analysis of biosynthesized Ds-AgNPs revealed that Ds-AgNPs exhibited very strong signal of silver (Fig. 5b), oxygen and weak signals Si and chlorine peaks, indicating the complete reduction of silver ions to elemental silver. Likewise, the previous reports on silver nanoparticles revealed similar results in EDX analysis. The Ds-AgNPs showed high emission energy at 3 keV for silver and insubstantial signals for other elements.

#### Atomic force microscopy analysis (AFM) analysis of biosynthesized Ds-AgNPs

**Figure 6:**
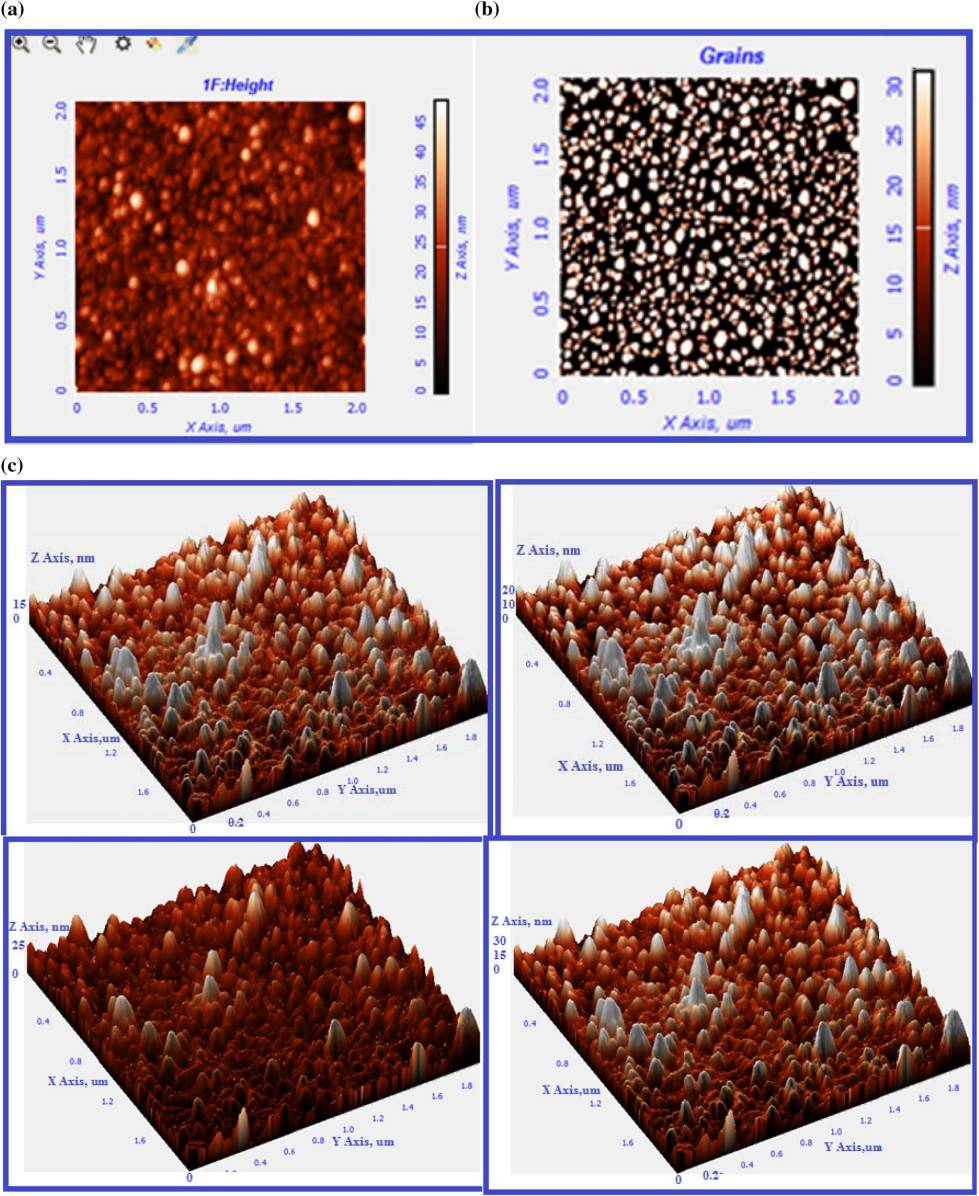
(**a**) 2D image of biosynthesized Ds-AgNPs. (**b**) Grains detected. (**c**) 3D image of biosynthesized Ds-AgNPs. Grains analysis of Ds-AgNPs by Z coloration method.

The AFM analysis of biosynthesized Ds-AgNPs was done to detect the surface morphology and topology of nanoparticles. The results indicated that the Ds-AgNPs are spherical in shape and the size of the Ds-AgNPs were in the range of 5 nm to 30 nm and the average grain size detected as 23 ± 5 nm (Fig. 6a,b). Further, we have also carried out Z-coloration analysis with 3D image of Ds-AgNPs to find out the distribution of different size of biosynthesized Ds-AgNPs. The results clearly indicate that the Ds-AgNPs are in the range of 5 nm to 30 nm (Fig. 6c), It is concluded that the size of Ds-AgNPs almost similar to that of Particle size analysis and TEM analysis.

### In vitro antioxidant activity of Ds-AgNPs by DPPH, H_2_O_2_ and nitric oxide (NO) methods

**Figure 7:**
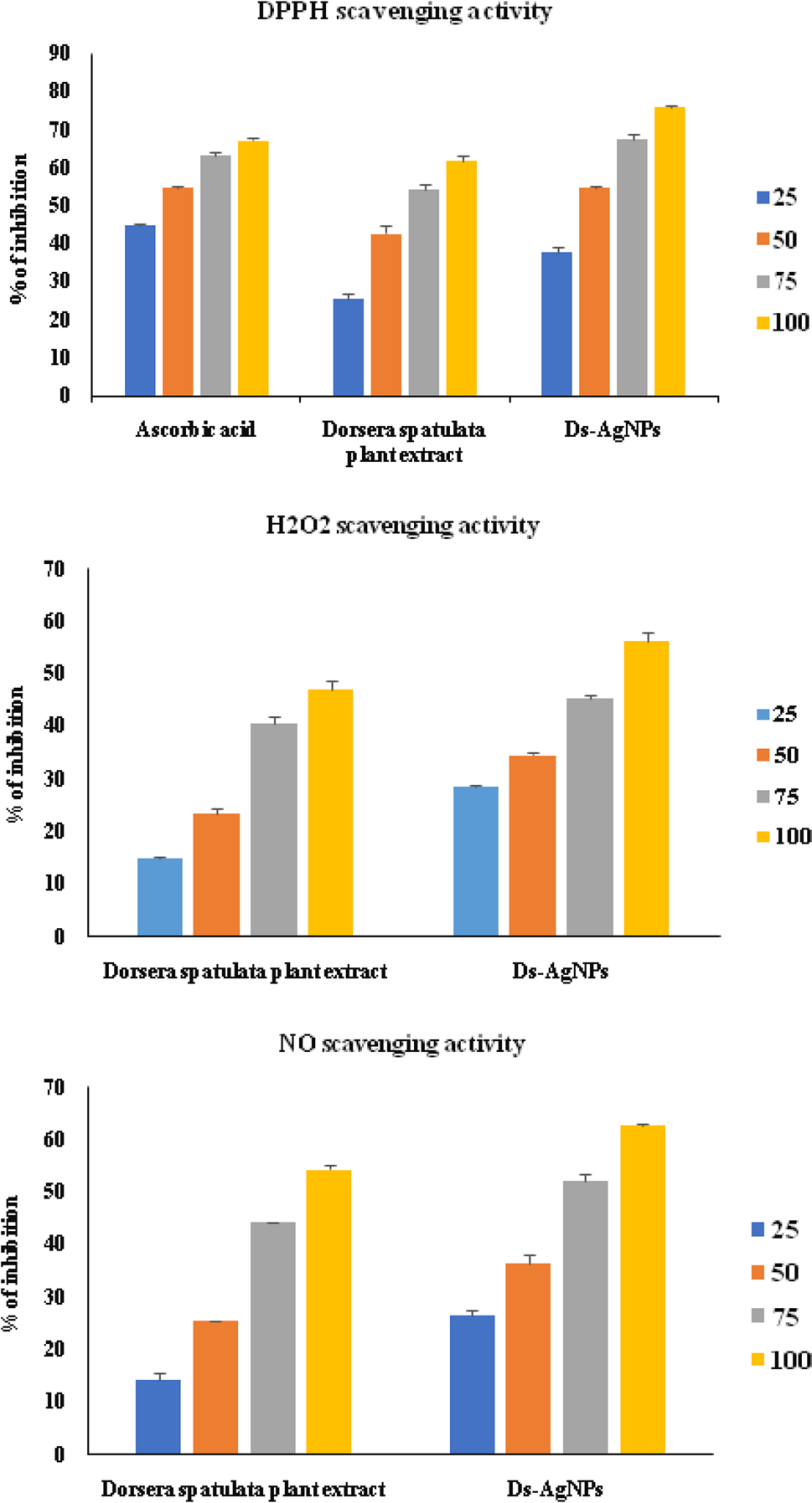
(**a**) DPPH Scavenging activity. (**b**) H_2_O_2_ Scavenging activity. (**c**) NO Scavenging activity of biosynthesized Ds-AgNPs.

In the present study antioxidant activity of *D. spatulata *var. bakoensis plant extract (Ds-PE) and Ds-AgNPs was determined by three important assays namely DPPH assay, H_2_O_2_ assay and NO scavenging assays. The results were shown in Table 1(i) and Fig. 7a DPPH assay (Table 1(ii) and Fig. 7b) H_2_O_2_ free radicals assay and Table 1(iii) and Fig. 7c Nitric oxide assay for both the *D. spatulata *var. bakoensis plant extract (Ds-PE) and biosynthesized Ds-AgNPs. The results revealed that DPPH assay is the best antioxidant assay with utmost inhibition of free radicals at the percentage of 62% and 76% for both (Ds-PE) and Ds-AgNPs, with an IC_50_ values calculated at the highest concentration of 100 µg/mL is as follows 42.2 µg/mL for DS-PE) and 29.27 µg/mL for (Ds-AgNPs). Similarly, the H_2_O_2_ antioxidant assay reveals that both the DsPE and Ds-AgNPs show good scavenging activity of 47% and 56% against H_2_O_2_ radicals with an IC_50_ values 45.25 µg/mL and 35.2 µg/mL respectively for Ds-PE and Ds-AgNPs. Lastly in NO free radical assay, DsPE and Ds-AgNPs exhibited very good scavenging activity of 54.4% and 62.78% against NO free radicals, with an IC_50_ values of 48.4 µg/mL and 36.2 µg/mL. From the above results it is clearly understood that the antioxidant activity was dose dependent manner in all the three methods, and the data revealed that the DPPH showed superior antioxidant activity when compared with other two methods, followed by NO free radical assay and H_2_O_2_ antioxidant activity. Finally, it is concluded that efficient DPPH scavenging activity of Ds-AgNPs could be due to the proteins, polyphenols and flavonoids which are present plant extract. Proteins, alcohols, and Phenols might have actively participated in the green synthesis of Ds-AgNPs. Proteins might be involved in the coating and capping of the Ds-AgNPs, because the *D. spatulata *var. bakoensis plant is an insectivorous plant which is rich in proteins and other bioactive compounds.

**Table 1:** In vitro antioxidant activity of Ds-PE and Ds-AgNPs by DPPH method, H_2_O_2_ method and Nitric oxide (NO) method.

DPPH method	Radical scavenging activity ± SD (%)				Ic50
Sample name	25 µg/mL	50 µg/mL	75 µg/mL	100 µg/mL	
**(i) In vitro antioxidant activity by DPPH method**					
Ascorbic acid	45.07 ± 0.26	54.73 ± 0.07	63.54 ± 0.56	67.43 ± 0.67	33.93 ± 0.12
*Dorsera spatulata* plant extract	25.45 ± 1.52	42.86 ± 1.96	54.48 ± 1.36	62.10 ± 1.34	42.29 ± 1.31
Ds-AgNPs	37.81 ± 1.45	54.91 ± 0.36	67.64 ± 1.47	76.20 ± 0.39	29.27 ± 0.63

H_2_O_2_ method	Radical scavenging activity ± SD (%)				Ic50
Sample name	25 µg/mL	50 µg/mL	75 µg/mL	100 µg/mL	
**(ii) In vitro antioxidant activity by H**_**2**_**O**_**2**_** method**					
*Dorsera spatulata* plant extract	15.14 ± 0.34	23.4 ± 1.24	40.56 ± 1.32	47.02 ± 1.76	45 ± 1.25
Ds-AgNPs	28.64 ± 0.25	34.64 ± 0.6	45.34 ± 0.56	56.43 ± 1.57	35.2 ± 0.48

Nitric oxide (NO) method	Radical scavenging activity ± SD (%)				Ic50
Sample name	25 µg/mL	50 µg/mL	75 µg/mL	100 µg/mL	
**(iii) In vitro antioxidant activity by nitric oxide (NO) method**					
*Dorsera spatulata* plant extract	14.26 ± 1.4	25.6 ± 0.14	44.28 ± 0.2	54.44 ± 0.67	48.4 ± 0.16
Ds-AgNPs	26.58 ± 1.12	36.62 ± 1.48	52.18 ± 1.2	62.78 ± 0.22	36 ± 01.26

### Antimicrobial activity of biosynthesized Ds-AgNPs

#### Antibacterial activity

**Figure 8:**
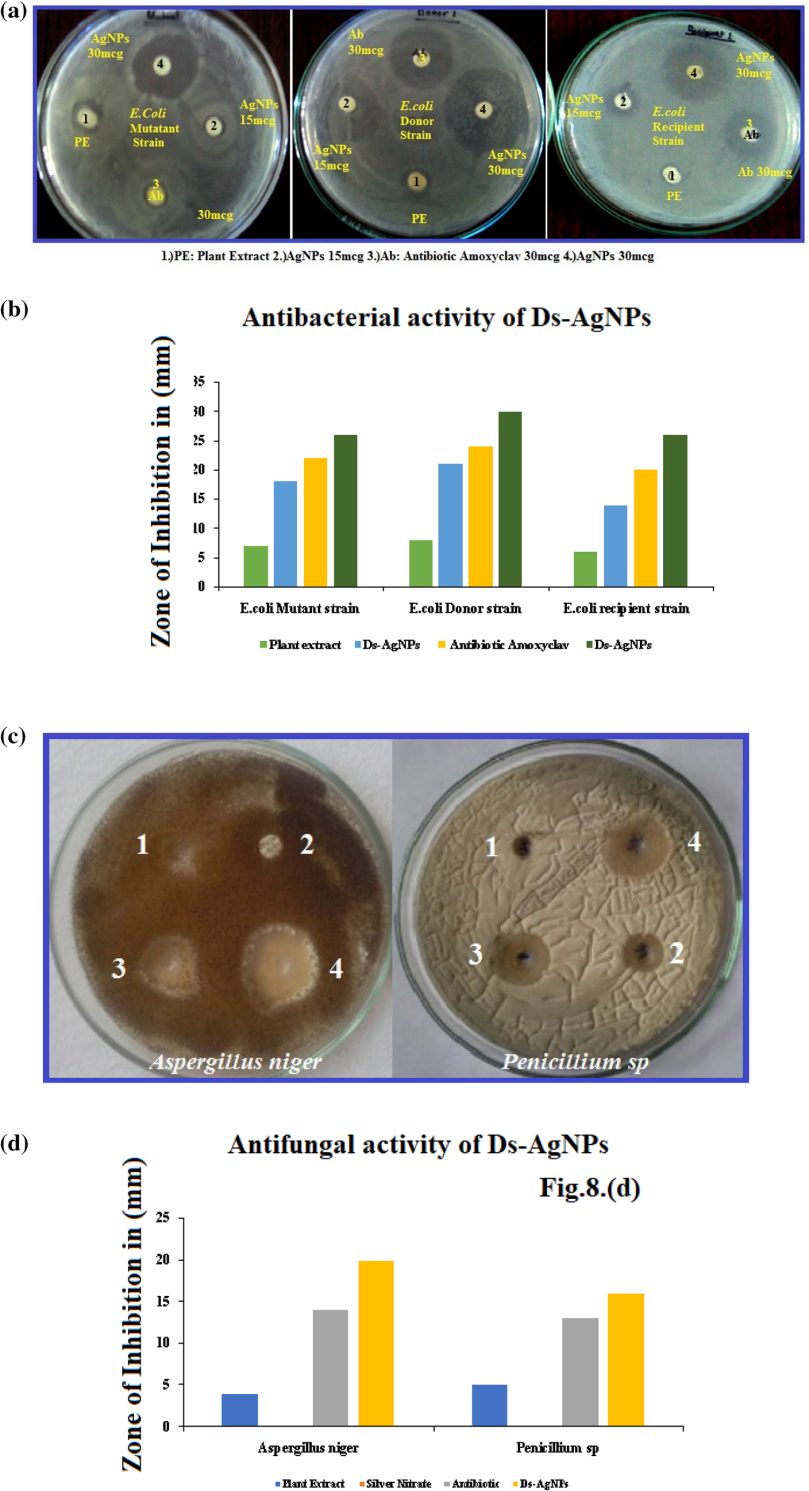
(**a**) Antibacterial activity of AgNPs on Gram negative antibiotic resistant *E. coli* strains, *E. coli* Mutant strain, *E. coli* Donor strain, *E. coli* recipient strain. (**b**) Graphical analysis of Antibacterial activity of Ds-AgNPs on Gram negative antibiotic resistant *E. coli* strains *E. coli* Mutant strain, *E. coli* Donor strain, *E. coli* recipient strain. (**c**) Antifungal activity of AgNPs on *Aspergillus niger* and *Penicillium* sp. *Aspergillus niger*: (1) plant extract, (2) silver nitrate, (3) nystatin antibiotic (25mcg), (4) Ds-AgNPs. *Penicillium* sp.: (1) silver nitrate, (2) plant extract, (3) nystatin antibiotic (25mcg), (4) Ds-AgNPs. (**d**) Graphical representation of Antifungal activity of Ds-AgNPs on *Aspergillus niger* and *Penicillium* sp.

The antibacterial studies of the biosynthesized Ds-AgNPs was carried out by using three different strains antibiotic resistant gram negative *Escherichia coli*. The strains are as follows *E. coli* mutant strain, *E. coli* donor strain and *E. coli* recipient strain. The Ds-AgNPs *were* found have very efficient antibacterial activity against the three *Escherichia coli* bacterial strains at the concentration of 30 μl (30mcg). The zone of inhibition (ZOI) of Ds-AgNPs with different concentration of 15 mcg and 30 mcg were reported in Table 2, by comparing along with standard antibiotic viz. Amoxyclav (Himedia SD063) and plant extract. The ZOI of Amoxyclav (Himedia SD063) in all three strains of antibiotic resistant *E. coli* mutant strain, *E. coli* donor strain and *E. coli* recipient strain were 22 mm, 24 mm and 20 mm, whereas the ZOI of Ds-AgNPs at the concentration of 30 mcg were 26 mm, 30 mm and 26 mm (Fig. 8a,b). So, it is clearly understood that the Ds-AgNPs comprise higher inhibitory activity then the standard antibiotic Amoxyclav. Though there are several reports on antimicrobial activity of silver nanoparticles which were bio-fabricated or green synthesized by different parts of plant materials, revealed that in most of the cases the plant extracts show negligible amount of antimicrobial activity or no inhibition of bacteria, while the biosynthesized AgNPs revealed enhanced and exceptional antimicrobial activity. In the present study also, the Ds-PE showed minimum inhibition zones of 07 mm, 08 mm, and 06 mm respectively, but the biosynthesized Ds-AgNPs revealed superior antibacterial activity when compared to Ds-PE alone.

**Table 2:** Antibacterial activity of Ds-AgNPs on antibiotic resistant *E. coli.*

Samples	1. Plant extract	2.Ds-AgNPs 15mcg	3. Antibiotic amoxyclav 30 mcg	4. Ds-AgNPs 30 mcg
**Zone of inhibition**				
*E. coli* mutant strain	7 mm	18 mm	22 nm	26 mm
*E. coli* donor strain	8 mm	21 mm	24 mm	30 mm
*E. coli* recipient strain	6 mm	14 mm	20 mm	26 mm

When the bacterial cells are treated with AgNPs, which are in nano-size particles will come in contact with bacterial cell wall and cause cell damage by penetrating into the cell wall due to electrostatic forces and endocytosis, subsequently cause bacterial cell death by production of reactive oxygen species which cause inflammation, lead to DNA damage and other cell organelles, it’s also depends upon the concentration of silver nanoparticles used for the assay, higher the concentration of AgNPs superior zone of inhibition was observed by various researchers. Similar type of results was observed AgNPs biosynthesized with turmeric extracts^41^. Other recent study by root extract of *Salvadora persica* mediated silver nanparticles, also reveals that ZOI will also depend upon the size of the nanoparticles and concentration of nanoparticles. Smaller the size of nanoparticles more easily the nanoparticles will percolate through the cell membrane and induce toxicity and cause cell death in both gram positive *Staphylococcus epidermidis* and gram negative *Escherichia coli* bacteria^42^. In the past Kim et al. and Feng et al.also studied the antibacterial efficacy of silver nanocomposite hydrogels and mechanistic effect of AgNPs against *Escherichia coli and Staphylococcus aureus* also reveals similar results in which protein synthesis is arrested due AgNPs^43,44^. In another study silver nanoparticles synthesized using *Cynodon dactylon* leaves also revealed that, the AgNPs will reacts with proteins of bacterial wall and penetrate to cells and cause DNA damage and inhibit DNA replication^45^. Erick et al.also investigate antibacterial activity against both Gram + ve and Gram-ve bacteria in an dose dependent manner^46^.

#### Antifungal activity of Ds-AgNPs

The biosynthesized Ds-AgNPs have proved to be exceptionally lethal against two fungal species *Aspergillus niger* and *Penicillium *sp. at the concentration of 25mcg (Fig. 8c). The outcome of the inhibitory activity of biosynthesized Ds-AgNPs were tabulated in (Table 3) and shown in (Fig. 8d) along with the reference drug Nystatin, (SD025, HiMedia). The biosynthesized Ds-AgNPs showed excellent ZOI against *Aspergillus niger* is 20 mm and 16 mm *Penicillium *sp., whereas ZOI of standard drug Nystatin, (SD025, HiMedia) against *Aspergillus niger* and *Penicillium *sp. were 14 mm and 13 mm in diameter. The ZOI of plant extract of *D. spatulata *var. bakoensis were 4 mm and 5 mm and 0.002 M silver nitrate solution did not show any inhibition (Table 3), from the above results, it is concluded that the biosynthesized Ds-AgNPs have excellent antifungal activity than the standard antifungal drug Nystatin. Therefore, the biosynthesized Ds-AgNPs can be useful as effective and excellent antifungal agent and can be widely used in pharmaceutical industries for the development of antifungal ointments and other formulations^47^. Similar type of results were reported recently by seed extract mediated AgNPs by *Plantago major*, in which the silver nanoparticles revealed good antifungal activity against *Penicillium *sp. and also good antibacterial activity against *E. coli* also^48^.

**Table 3:** Antifungal activity of Ds-AgNPs on *Aspergillus niger* and *Penicillium* sp.

	Plant extract (20mcg)	Silver nitrate (20mcg)	Antibiotic (nystatin 20mcg)	Ds-AgNPs (20mcg)
*Aspergillus niger*	4 mm	Nil	14 mm	20 mm
*Penicillium* sp.	5 mm	Nil	13 mm	16 mm

### Anticancer activity of biosynthesized *Ds*-AgNPs

The ability of NPs to make a way into cells primarily depends on the physiochemical properties including shape, size and surface net charge. In the current study, the size of biosynthesized Ds-AgNPs is in-between 12 and 40 nm in size with average size of 21.9 nm. It is well known fact that the nanoparticles having the size below 100 nm are said to be of biomedical importance. Similarly, the nanoparticles having size < 50 nm are said be very efficient in anticancer activity because of their minute size they can easily taken up and penetrate deeply into the cancer cells by endocytosis subsequently and cause cell death or apoptosis and eradicate tumorogenic cells. Whereas the nanoparticle having size > 50 nm cannot penetrate easily in the cancer cell and cannot completely damage the cancer cells or tumorogenic cells. So it is concluded that nanoparticles of minute size have three important properties by which they can easily diffuse, deep infiltration and improved accumulation can results in complete eradication of cancer cells or tumorogenic cells^49^.

**Figure 9:**
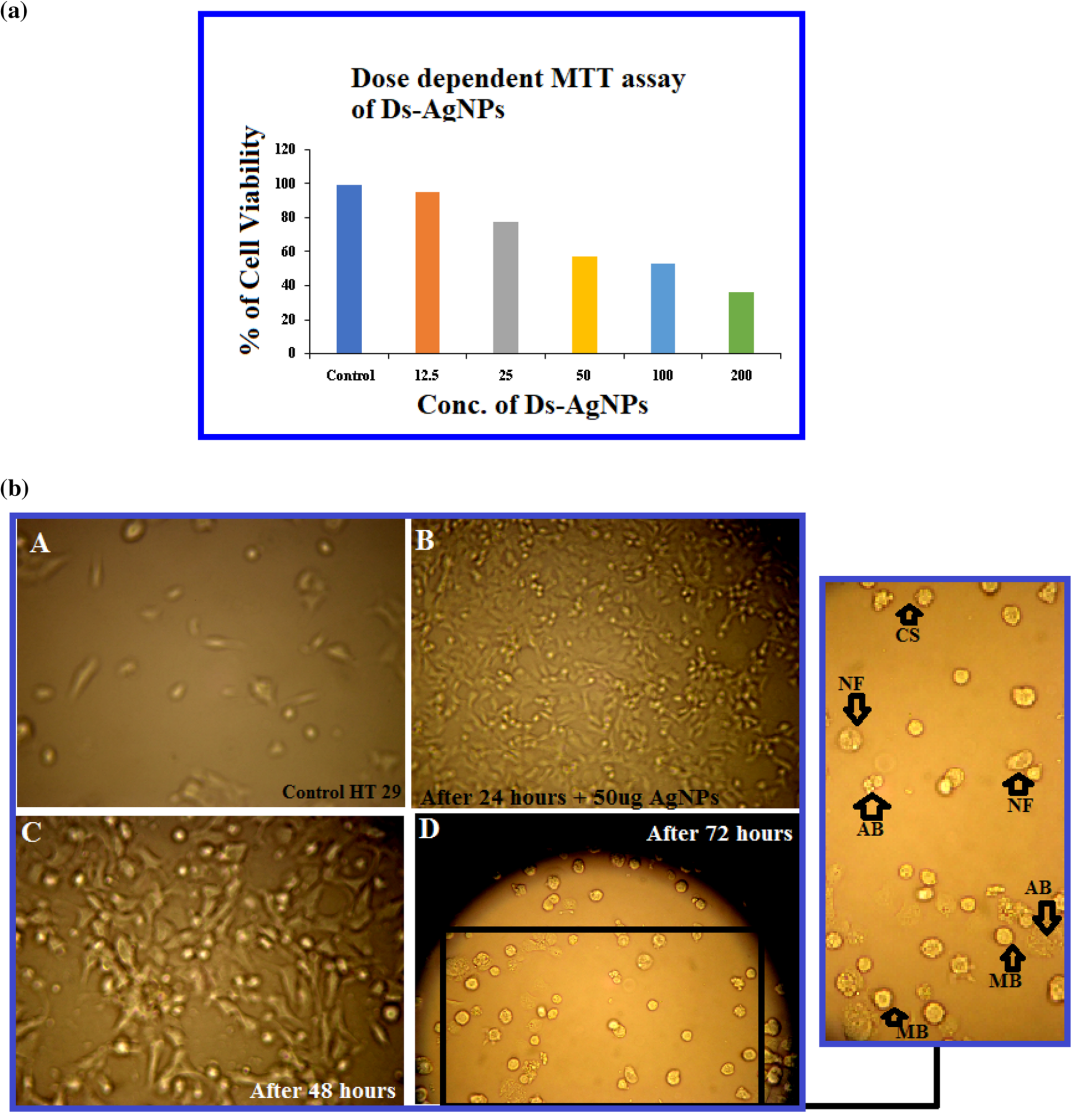
(**a**) Dose dependent Cytotoxic effect of Ds-AgNPs on HT-29 human colon cancer cell by MTT assay. (**b**) Cytotoxic effect of AgNPs on HT-29 human colon cancer cells and its Morphological changes of HT-29 cells observed under an inverted light microscope. (A) Untreated HT-29 cells. (B) HT-29 cells t0reated with 50ug of AgNPs after 24 h. (C) HT-29 cells treated with 50ug of AgNPs after 48 h. (D) HT-29 cells treated with 50ug of AgNPs after 72 h. After 72 h the cells showed distinctive characteristics of apoptosis, such as apoptotic bodies (AB), cellular shrinkage (CS), nuclear compaction (NC), membrane blebbing (MB), and nuclear fragmentation (NF) (magnification ×100).

In the present study, anticancer activity of the Ds-AgNPs was evaluated against HT29 human colon cancer cells by MTT assay. The results revealed that the Ds-AgNPs induced cytotoxicity in HT29 cancer cells by concentration dependent approach (Fig. 9a). Higher the concentration of Ds-AgNPs decreases the cell viability of cancer cells. Ds-AgNPs showed maximum inhibition of 92% against HT29 colon cancer cells. IC_50_ values of the Ds-AgNPs were found to be 52.81 µg/mL against HT29 colon cancer cells. The (Fig. 9b) reveals the cytotoxic effects of Ds-AgNPs at concentration of 50 µg/mL which almost equivalent IC_50_ value at different time interval up to 72 h to observe the morphological changes in the cancer cell due cytotoxic effect of the biosynthesized Ds-AgNPs. It is clearly evident that the HT 29 cancer cells completely dead after 72 h no viable cells were observed, which proves the efficacy of biosynthesized Ds-AgNPs as an anticancer agent this may due to the minute size of Ds-AgNPs. Recently, Alqahtani et al. also reported efficacy of biosynthesized AgNPs by lichens on HCT-116 (Human colorectal cancer cell) and MDA-MB-21 (Breast cancer cell) and FaDU (Pharynx cancer cell) by *in vitro* cytotoxic MTT assay also revealed similar results to our present study which is size and dose dependent^50^. Kabir et al. also conducted cytotoxic studies in different cancer cell lines which were similar to the above results^51^, an another study by photosynthesized AgNPs using *Ficus religiosa* also proved have substantial cytotoxicity against COLO205 cells^52^, other study using silver nanoparticles by *M. koenigii* have also proven to an effective applicable drug for colon cancer^53^. Finally it could be concluded that the results of cytotoxic activity of biosynthesized Ds-AgNPs are in line with previous reports and any difference in cytotoxic assay results could vary due size, shape and concentration and different cancer cell lines.

## Summary and conclusion

In this report it was demonstrated the biofabrication of nanosized Ds-AgNPs using insectivorous plant *Drosera spatulata *Labill var. bakoensis extract, being the first of its kind. The biosynthesized Ds-AgNPs are spherical, well-dispersed with average range size of 10 to 20 nm and exhibited outstanding antimicrobial activities against clinical bacteria and fungi isolates. Furthermore, Ds-AgNPs also demonstrated superior free radical scavenging activities and strong cytotoxic potential against HT-29 colon cancer cell lines, indicating the possibility of employing and developing Ds-AgNPs as multifaceted therapeutic agents for various clinical conditions (Supplementary Information).

## Supplementary Information

Supplementary Information.
