# Supplementary material for: Multifaceted phytogenic silver nanoparticles by an insectivorous plant Drosera spatulata Labill var. bakoensis and its potential therapeutic applications
Source: Sci Rep. 2021 Nov 9;11:21969. doi: 10.1038/s41598-021-01281-8 (PMC8578548; doi:10.1038/s41598-021-01281-8)

The present work is represented in a schematic diagram (S1)


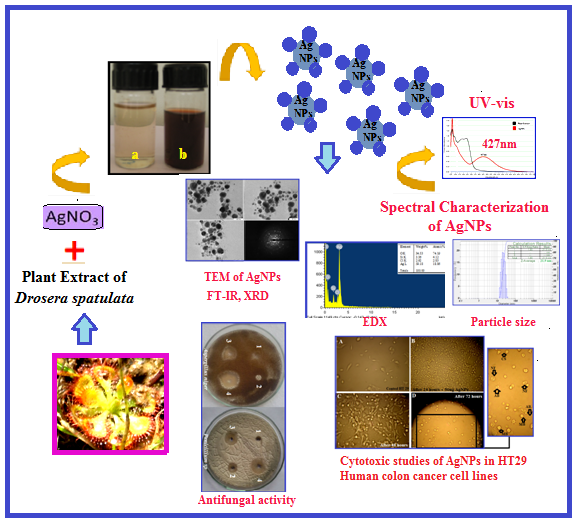


**S1:** Schematic diagram of biosynthesis of **s**ilver **n**anoparticles (AgNPs), the spectral characterization and biomedical applications

The roles of plant extract and Ds-AgNPs in antioxidant activity have been shown in schematic diagram **S.2.** below


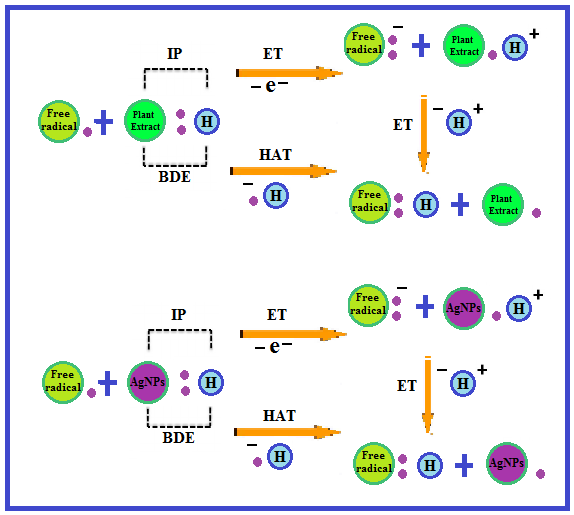


**S.2. Schematic diagram of Free radical scavenging activity of Plant extract and biofabricated Ds-AgNPs**


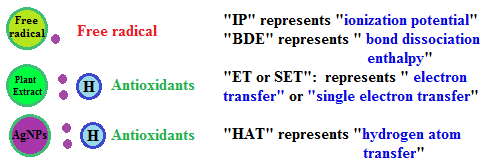


The mode of action of Ds-AgNPs was illustrated in a schematic diagram S.3. as follows; AgNPs binds with ergosterol which is a major constituent of fungal cell membrane, resulting in creation of small pores in the membrane, which causes and promotes the loss of sodium, potassium and hydrogen ions and other cellular constituents like sugars and amino acids. These subsequently blocks the several membranes bound enzymes which can further cause the death of fungal cells.


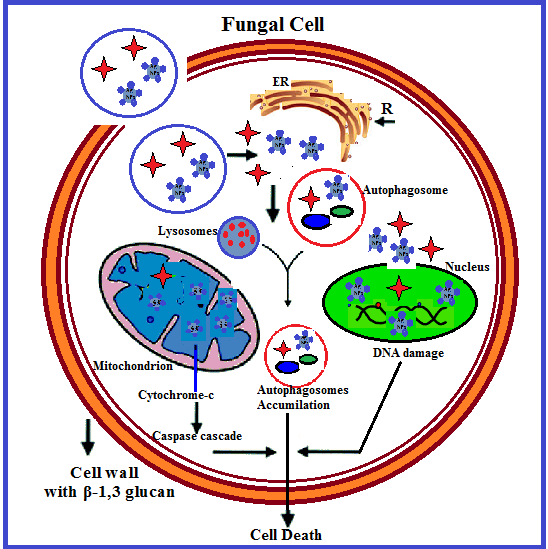


**S.3. Schematic diagram of Antifungal Activity of biosynthesized Ds- AgNPs**


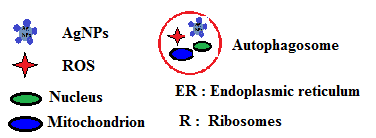

Supplement: Supplementary file 1 — Supplementary Information. [file 41598_2021_1281_MOESM1_ESM.docx]
